# Evaluation of a decellularized bronchial patch transplant in a porcine model

**DOI:** 10.1038/s41598-023-48643-y

**Published:** 2023-12-08

**Authors:** Daisuke Taniguchi, Satoshi Kamata, Sara Rostami, Stephen Tuin, Alba Marin-Araujo, Kelly Guthrie, Thomas Petersen, Thomas K. Waddell, Golnaz Karoubi, Shaf Keshavjee, Siba Haykal

**Affiliations:** 1 Latner Thoracic Research Laboratories, Division of Thoracic Surgery, Toronto General Hospital Research Institute, University Health Network, 200 Elizabeth Street suite 8N-869, Toronto, ON M5G2C4 Canada; 2 Department of Surgical Oncology, Nagasaki University Graduate School of Biomedical Sciences, 1-7-1 Sakamoto, Nagasaki, 852-8501 Japan; 3 United Therapeutics Corp, Research Triangle Park, NC 27709 USA; 4 Department of Mechanical and Industrial Engineering, University of Toronto, Toronto, Canada; 5 Department of Laboratory Medicine and Pathobiology, University of Toronto, Toronto, Canada; 6 Institute of Biomaterials and Biomedical Engineering, University of Toronto, Toronto, Canada; 7 Division of Plastic & Reconstructive Surgery, University Health Network, University of Toronto, Toronto, ON Canada; 8 Institute of Medical Sciences, University of Toronto, Toronto, ON Canada

**Keywords:** Biological techniques, Biological models, Immunological models

## Abstract

Biological scaffolds for airway reconstruction are an important clinical need and have been extensively investigated experimentally and clinically, but without uniform success. In this study, we evaluated the use of a decellularized bronchus graft for airway reconstruction. Decellularized left bronchi were procured from decellularized porcine lungs and utilized as grafts for airway patch transplantation. A tracheal window was created and the decellularized bronchus was transplanted into the defect in a porcine model. Animals were euthanized at 7 days, 1 month, and 2 months post-operatively. Histological analysis, immunohistochemistry, scanning electron microscopy, and strength tests were conducted in order to evaluate epithelialization, inflammation, and physical strength of the graft. All pigs recovered from general anesthesia and survived without airway obstruction until the planned euthanasia timepoint. Histological and electron microscopy analyses revealed that the decellularized bronchus graft was well integrated with native tissue and covered by an epithelial layer at 1 month. Immunostaining of the decellularized bronchus graft was positive for CD31 and no difference was observed with immune markers (CD3, CD11b, myeloperoxidase) at two months. Although not significant, tensile strength was decreased after one month, but recovered by two months. Decellularized bronchial grafts show promising results for airway patch reconstruction in a porcine model. Revascularization and re-epithelialization were observed and the immunological reaction was comparable with the autografts. This approach is clinically relevant and could potentially be utilized for future applications for tracheal replacement.

## Introduction

Tracheal reconstruction for airway injuries secondary to trauma, cancer and stenosis is required. Current replacement techniques lead to inadequate re-epithelialization, tracheal collapse and eventual failure^1–4^. Airway reconstruction is complex and challenging because of difficulties in achieving revascularization due to the anatomical features of segmental blood supply, risk of infection due to continuous contact with the external environment and the possibility of rejection of airway transplantation^4–6^. To overcome these limitations, the use of artificial airways has been extensively investigated^1,7^, however, no method is yet widely applicable for clinical treatment.

Tissue engineering and regenerative medicine approaches present a possible solution for airway reconstruction. A variety of attempts have been made to achieve appropriate airway regeneration using cell-free artificial prostheses^8–11^, biomaterials^12–14^, and decellularized tissues^15–18^. Decellularization technology allows for retention of mechanical scaffold properties, therefore offering a potentially promising basis for airway reconstruction^1,16,17,19–21^.

To test various decellularized materials and their ability for long-term tissue integration, a tracheal patch implant model was used and has been shown to be promising^18,22^. This approach has been used to evaluate regenerated airway grafts with pluripotent stem cells^23^. This model serves as the initial step to testing the long-term integration and strength of decellularized products. We have generated a decellularized pig bronchus from a decellularized lung and used it to create a tracheal patch to evaluate long-term integration, cell infiltration and revascularization in a tracheal transplantation model.

This large animal airway patch model could represent a proof of concept for the evaluation of re-epithelialization and long-term outcomes of a decellularized bronchial patch.

## Materials and methods

### Study design

**Figure 1:**
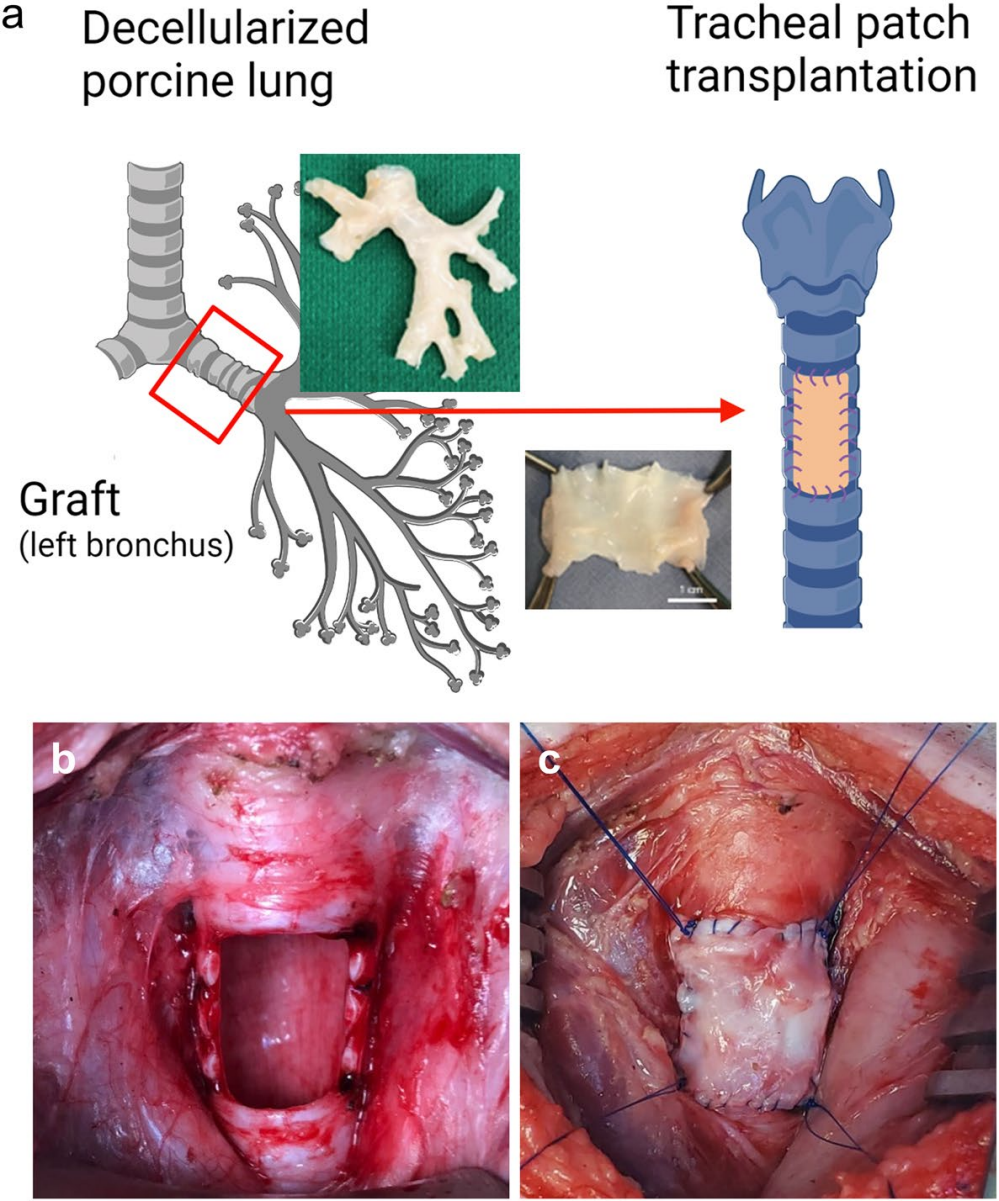
Left bronchus from decellularized porcine lung product served as a tracheal patch (**a**). A tracheal window was created in the recipient pig and transplanted (**b**). The decellularized bronchus patch was transplanted in the experimental group (**c**) while the original native patch was re-sutured in the control cases.

In the control group (n = 9), a piece of front wall of trachea measuring 3 cm × 2 cm was excised from the native trachea and re-sutured into the defect as an autograft. In the experimental group (n = 9), a tracheal window of 3 cm × 2 cm was created, as in the control group. The left bronchus from a decellularized lung was cut open longitudinally and transplanted as a tracheal patch graft (Fig. 1) maintaining the same anterior wall orientation. Scheduled euthanasia occurred after the postoperative observation periods of 7 days (n = 6), 1 month (30 days, n = 6), and 2 months (60 days, n = 6).

### Animals

Male Yorkshire pigs (30–50 kg) were utilized for these studies. All studies were approved by the Institutional Animal Care and Use Committee (IACUC) of the University Health Network and Toronto General Hospital Research Institute. Humane care was provided to all animals in accordance to the “Principles of Laboratory Animal Care” defined by the National Society for Medical Research and the “Guide for the Care of Laboratory Animals” issued by the National Institutes of Health. Reporting of use of experimental animals in this study followed recommendations specified by the ARRIVE guidelines (Approval number: AUP6130.8).

### Lung decellularization

Left lungs of juvenile pigs were decellularized via instillation of solutions to the bronchus based upon previously published methodologies^24–26^. In brief, tissues were first washed with phosphate-buffered saline (PBS) containing antibiotic/antimycotic solution, followed by treatment with repeated instillations of 0.1% Triton-X 100, 2% sodium deoxycholate (SDC) and 1 M NaCl. Deionized water washes occurred between each solution to reduce residual solutes and cellular components prior to advancing to the next solution. Finally, the lung scaffold was washed with PBS and antibiotic/antimycotic solutions and stored at 4 °C until derivation of the bronchial patch for implant. H&E, DNA analysis and quantitative proteomics were performed on the product.

### Tracheal patch surgery and post-operative care

A tracheal patch measuring 3 cm × 2 cm was created from the bronchus obtained from a decellularized pig lung. Specifically, the left bronchus was excised from the surrounding tissue. The resected tubular left bronchus was opened at its posterior surface in order to be used as a patch (Fig. 1) for an anterior wall defect. The detailed protocol of the surgery has been described previously^23^. Briefly, pigs were anesthetized using an intramuscular (i.m.) injection of a mixture of ketamine (25 mg/kg), atropine (0.04 mg/kg), and midazolam (0.15 mg/kg) and intubated. A midline incision was made in the neck of the pigs, and the cervical trachea was exposed. The area corresponding to the three tracheal rings below the cricoid cartilage was dissected (approximately 3 × 2 cm, one-third of the tracheal trunk) to make a tracheal window. The tip of the endotracheal tube was placed proximal to the window created which was then advanced distally to maintain the mechanical ventilation during the anastomosis. In the experimental group, the decellularized bronchus patch was transplanted, while in the control group the native tracheal wall was re-implanted, using interrupted and continuous sutures with 4–0 prolene. These are nonbiodegradable sutures allowing us to determine the border of native tissue from the graft. A Valsalva was performed to ensure no air leaks. The tracheal window was covered by the left sternocleidomastoid muscle to enhance revascularization after the tracheal patch transplantation. Anesthesia was weaned gradually and the pigs were extubated under observation with monitoring of pulse oximetry, respiratory rate and pattern. Meloxicam (0.3 mg/kg) and SR buprenorphine (0.24 mg/kg) were administered before waking the animal from anesthesia. Ceftiofur (3 mg/kg) was given daily postoperatively for 3 days. Salbutamol (2–4 ug/kg nostril) was administered as a bronchodilator as needed. No immunosuppressant was given in this study. Animals were examined twice a day until the scheduled sacrifice date for clinical signs of inflammation and general health.

### Histological analysis

Samples were fixed with 10% formalin solution for 1 day and then embedded in paraffin blocks and sectioned at 5 µm. Hematoxylin–eosin (HE) staining was performed according to established protocols^21^. Slides were covered using a standard coverslip and scanned using an Aperio slide scanner (Leica Biosystems; Concord, ON, Canada).

### Immunohistochemistry

Samples were evaluated for Cytokeratin 5 + 8 (CK5/8 anti-mouse (AE1/AE3), 1:400, Dako, CA, USA), CD31 (CD 31 anti-rabbit (Polyclonal), 1:50, Abcam, ON, Canada), Ki67 (Ki67 anti-rabbit (Polyclonal), 1:5000, Abcam), myeloperoxidase (MPO, anti-Rabbit (Polyclonal), 1:4000, Dako), CD11b (CD 11b anti-Rabbit (EPR1344), 1:4000, Abcam), and CD3 (CD3 anti-Mouse (F7.2.38), 1:1000, Dako). Antigen retrieval was performed via heat induction in 1 × citrate buffer (Sigma, USA), followed by incubation in 3% H_2_O_2_ (v/v) at room temperature (5-min) and a PBS wash. Blocking was performed with normal horse serum 2.5% (v/v) for 30-min and the primary antibody was incubated overnight at 4 °C using the aforementioned dilutions. A 30-min biotinylated secondary antibody incubation was followed by a 5-min PBS wash, a 30-min incubation in RTU Vectastain™ ABC (avidin–biotin complex, Vector Laboratories, Canada) reagent, and another 5-min PBS wash. Then, 30µL of ImmPACT™ DAB Chromogen (Vector Laboratories, Canada) was diluted in 1 mL of ImmPACT™ DAB Dilutant and placed onto each slide. Samples were counterstained in hematoxylin solution, washed in tap water, dehydrated in xylene, cleared, and mounted.

### Uniaxial tensile testing

Uniaxial tensile testing was evaluated with a DMA 850 Dynamic Mechanical Analyzer (TA Instruments, New Castle, DE) fitted with a tension clamp. Rectangular specimens were loaded in the grips with a torque of three inch-pounds. An initial preload of 0.01 N was applied to remove slack and standardize initial loading. Samples were tested in rate control mode at a strain rate of 50 mm per minute until failure. Tensile properties were evaluated using TRIOS software (TA Instruments).

### Scanning electron microscopy

Specimens were dehydrated in preparation for SEM imaging through a series of ethanol and water solutions. Specimens with the lumen surface and a cross section of the implanted patches were then dehydrated in a critical point dryer and mounted on specimen holders. Specimens were sputter coated to 15 nm with a 60:40 gold:palladium blend. Specimens were imaged using a Hitachi S-4700 Cold Cathode Field Emission Scanning Electron Microscope (Hitachi, Tokyo) at an acceleration voltage of 2.0 kV.

### Statistical analysis

Data was reported as mean ± standard deviation (SD). All statistical analyses were performed using JMP Pro software (version 14.2.0; SAS Institute, Cary, NC). Comparisons among the groups were performed by analysis of variance with Tukey’s honestly significant difference test and 2-way ANOVA. Values of p < 0.05 were considered significant.

Data was reported as mean ± standard deviation (SD). All statistical analyses were performed using GraphPad Prism software (version 10.0.2; GraphPad Software, Boston, MA). Comparisons among the groups were performed using the Kruskal–Wallis test with Dunn’s post hoc test. Values of p < 0.05 were considered significant.

## Results

### Validation of the decellularization process

Characterization of decellularized scaffold parenchyma demonstrates dsDNA was reduced 95% in comparison to native lung (21 + /– 23 ng/mg vs 449 + /– 176 ng/mg). Additionally, initial characterization of bronchus isolated from decellularized lung scaffold demonstrates clearance of live cells (by histology) and a 73% reduction in cellular component content (actin, tubulin, and histone) as measured by quantitative proteomics. Furthermore, Decellularized bronchus was evaluated histologically by H&E staining, which showed well decellularized epithelial layer (Supplemental Fig. 1).

### Lack of stenosis and presence of tissue integration at 2 months post-transplant of decellularized bronchus in a tracheal patch model

**Figure 2:**
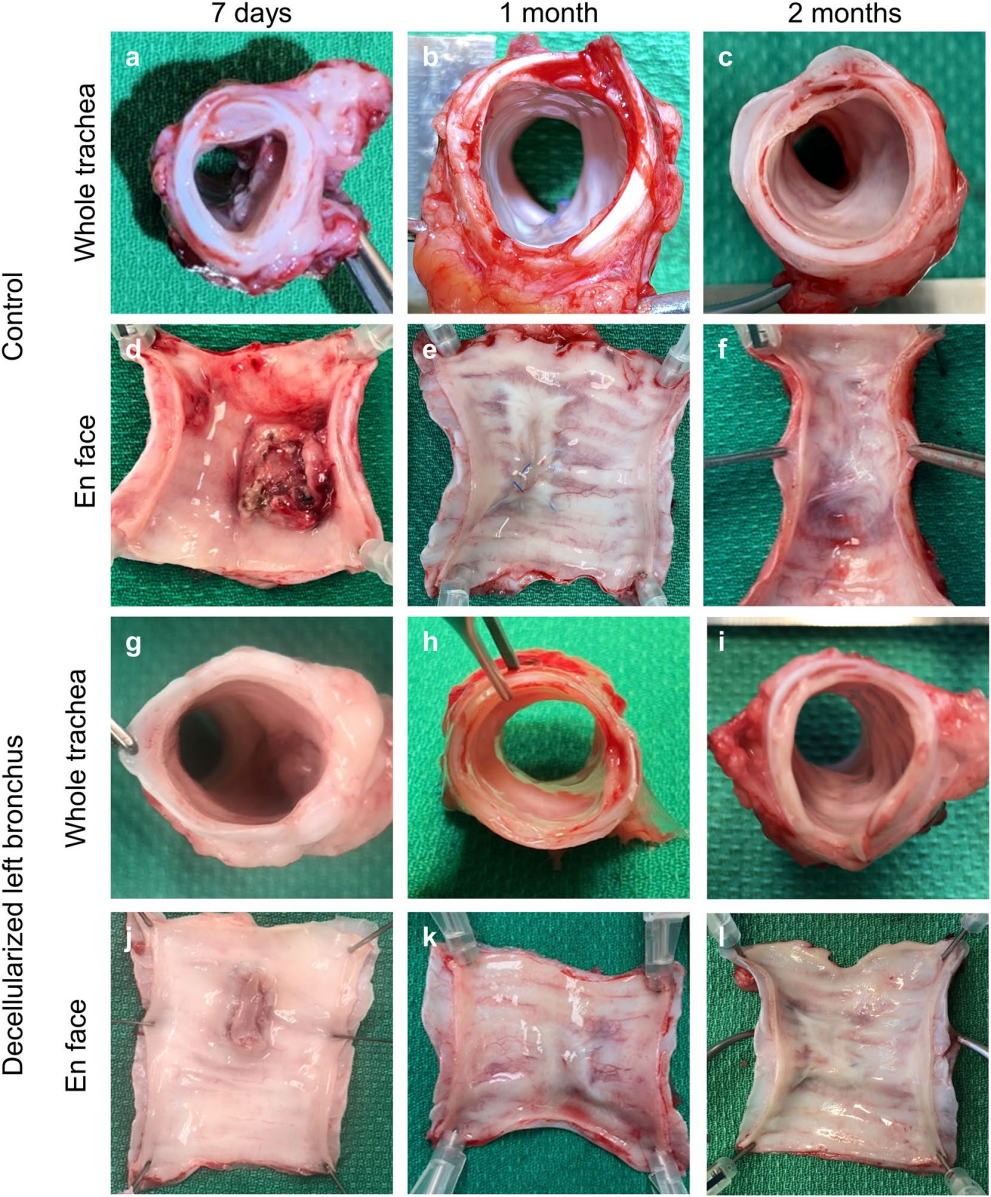
A decellularized bronchus graft served as a tracheal patch graft for up to 2 months. Macroscopic images of tracheal samples after the survival period. After 7 days post-operatively, granulation tissue was observed in the control group (**a**,**d**) in comparison to the decellularized group (**g**,**j**). After 1 month post-operatively, the grafts were well integrated with native tissue in both the control and experimental groups (**b**,**e**,**h**,**k**). A similar observation was found at 2 months post-operatively (**c**,**f**,**i**,**l**). None of the animals had severe stenosis or airway obstruction.

Tracheal patch transplant surgery with a decellularized porcine bronchus graft was a feasible approach (Fig. 2). All pigs recovered from general anesthesia and survived as planned without airway obstruction noted clinically. All pigs gained weight appropriately, with no differences noted between control and experimental groups. Macroscopic images of tracheal samples showed less granulation tissue in the experimental decellularized bronchus graft group than the control group after 7 days post-transplant (Fig. 2-a,d,g,j). At 1 month post-transplant (Fig. 2b,e,h,k) and 2 months post-transplant (Fig. 2c,f,i,l), the graft was well integrated with native tissue in both the control and experimental groups. None of the animals showed any signs of severe stenosis or obstruction of the airway (Fig. 2a–c,g–i).

### Integration of decellularized bronchus graft within native tissue and epithelial coverage

**Figure 3:**
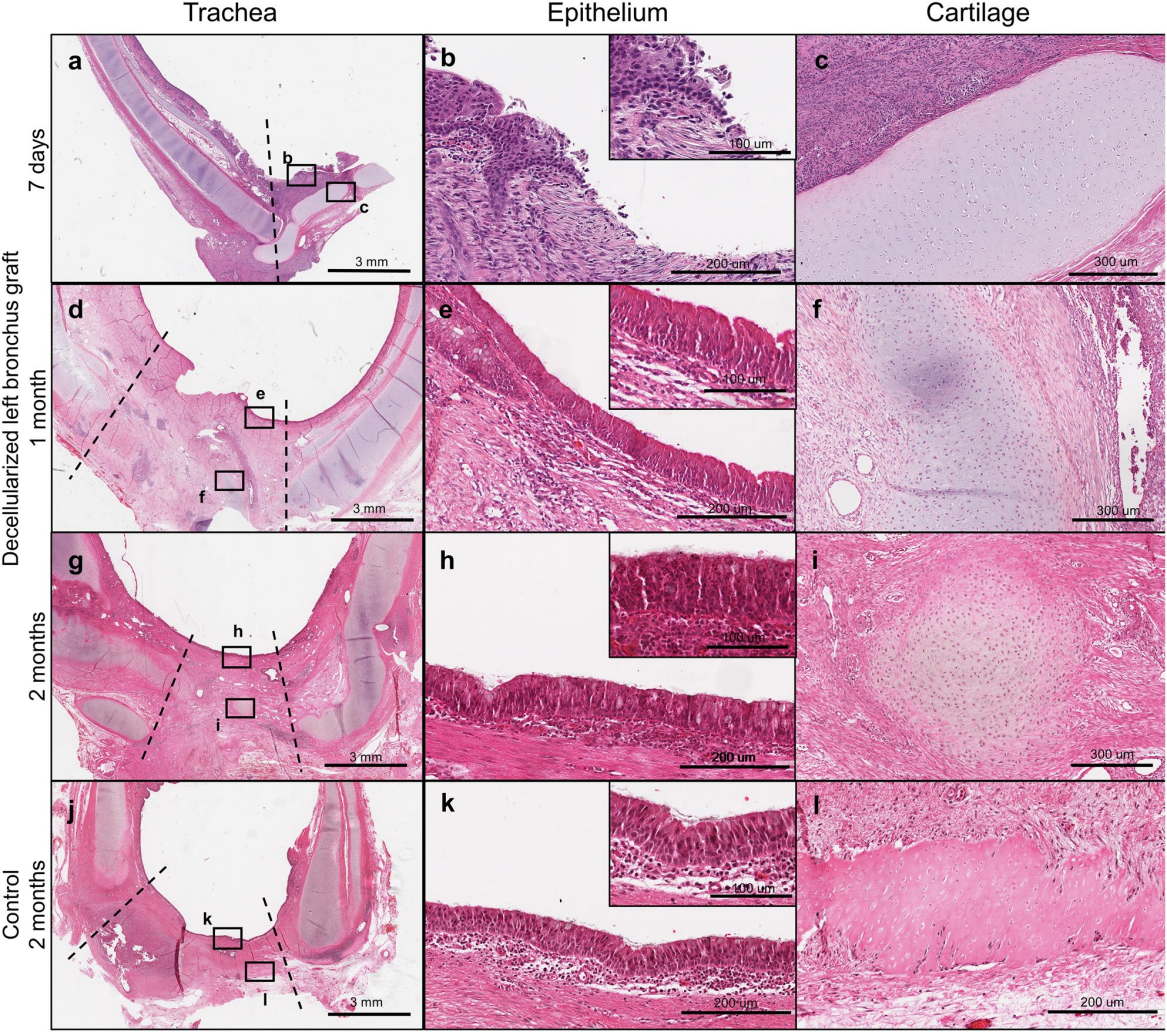
A decellularized bronchus graft was well integrated with the native tissue and covered by an epithelial layer. Hematoxylin–Eosin (HE) staining for 7 days survival cases (**a**–**c**), 1 month survival cases (**d**–**f**), 2 months survival cases (**g**–**i**). Integration of the graft was observed at 1 month and 2 months post-operatively. The histological morphology was comparable/similar with that of 2 months control cases (**j**,**k**). At 7 days post-operatively, re-epithelialization is incomplete (**b**). At 1 month post-operatively, full re-epithelialization is observed (**e**). At 2 months post-operatively, a robust pseudostratified ciliated epithelial layer was observed in both decellularized bronchus graft patch (**h**) and control (**k**) at the location of the patch. The integrity of the cartilage within the patch is observed at 7 days and 1 month (**c**,**f**) with some degradation at 2 months in both experimental and control cases (**i**,**l**). Dotted line represents the junction between the native tissue and the graft.

Histological assessment by H&E showed incomplete re-epithelialization of the decellularized tracheal patch at Day 7 (Figs. 3a,b). At 1 month post-transplant, full re-epithelialization was observed within the decellularized tracheal patch with an epithelium (Figs. 3d,e). A pseudostratified epithelium composed of ciliated and goblet cells was observed. This was also observed at 2 months post-transplant (Figs. 3g,h). These samples were compared to control cases at 2 months (Fig. 3j,k). Integrity of the cartilage within the graft was observed at 7 days and 1 month post-transplantation (Fig. 3c,f) with some degradation in both controls and experimental groups at 2 months (Fig. 3i,l). There appears to be retained cartilage in these experiments^15–17^.

### Re-epithelialization and re-vascularization of the decellularized bronchus graft

**Figure 4:**
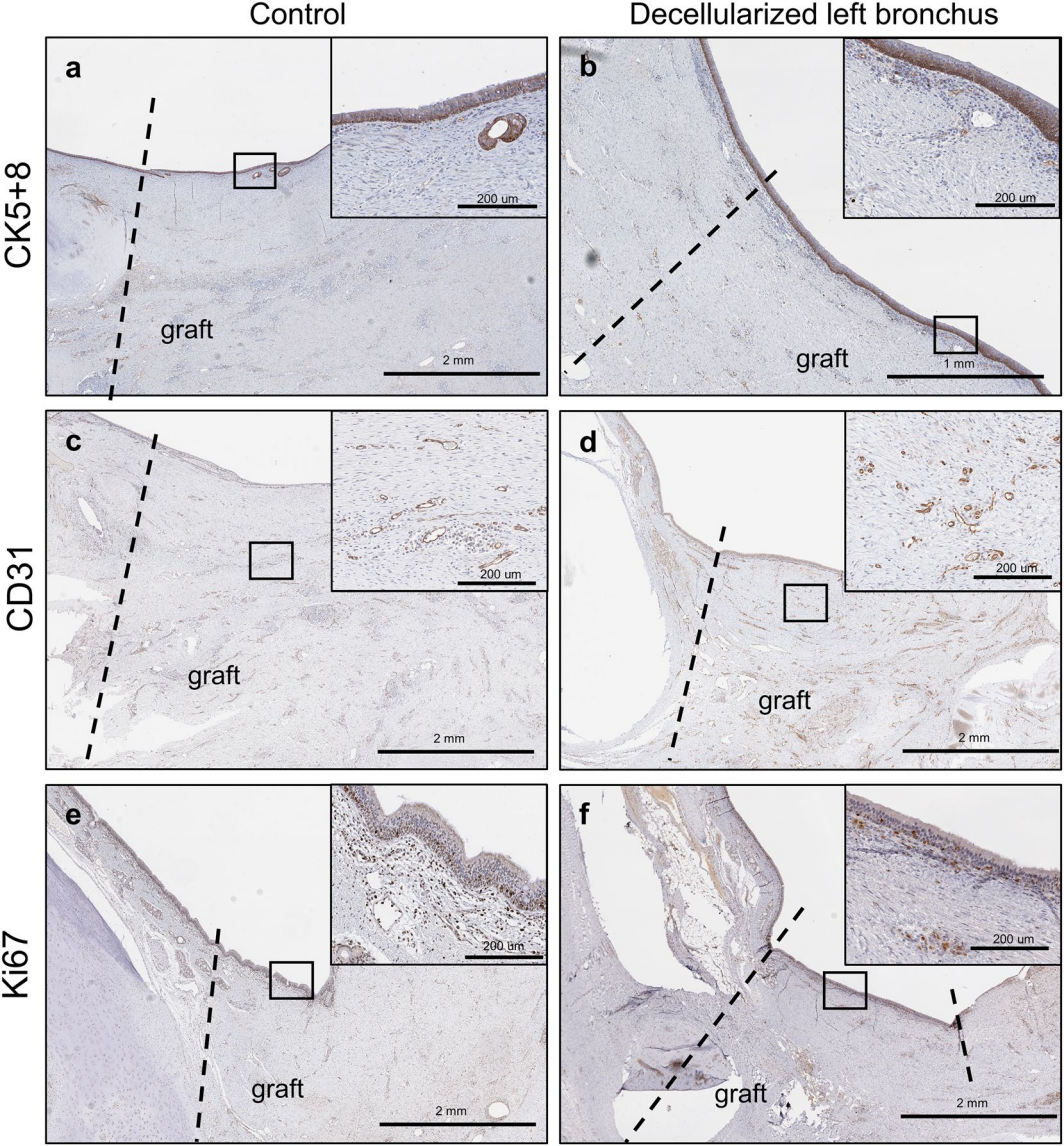
Re-epithelialization and revascularization of the decellularized tracheal patch. CK5 + 8 staining was observed within the graft in both control cases (**a**) and experimental cases (**b**) at 1 month post-operatively. Additionally, CD31 + staining was observed at 1 month post-operatively in both control cases (**c**) and experimental cases (**d**). Ki67 + staining shows no difference between the control cases (**e**) and the experimental cases (**f**) at 1 month post-operatively. Dotted line represents the junction between the native tissue and the graft.

CK5/8 immunohistochemistry staining showed presence of ciliated cells covering the entirety of the patch in both controls and decellularized bronchus at 1-month post-transplantation (Fig. 4a,b). Positive staining for CD31 was observed in both control cases and decellularized patch samples (Fig. 4c,d). Ki67 + immunohistochemistry staining also showed no difference between the control cases and the decellularized patch (Fig. 4e,f). SEM imaging revealed the presence of ciliated cells on the luminal surface of both the control patch and decellularized bronchus patch after 1 month (Fig. 6E,G). Cross sectional images of cartilage did not indicate any evidence of gross degradation after 1 month (Fig. 6D,F).

### Immunological profile in decellularized bronchus patch transplantation

**Figure 5:**
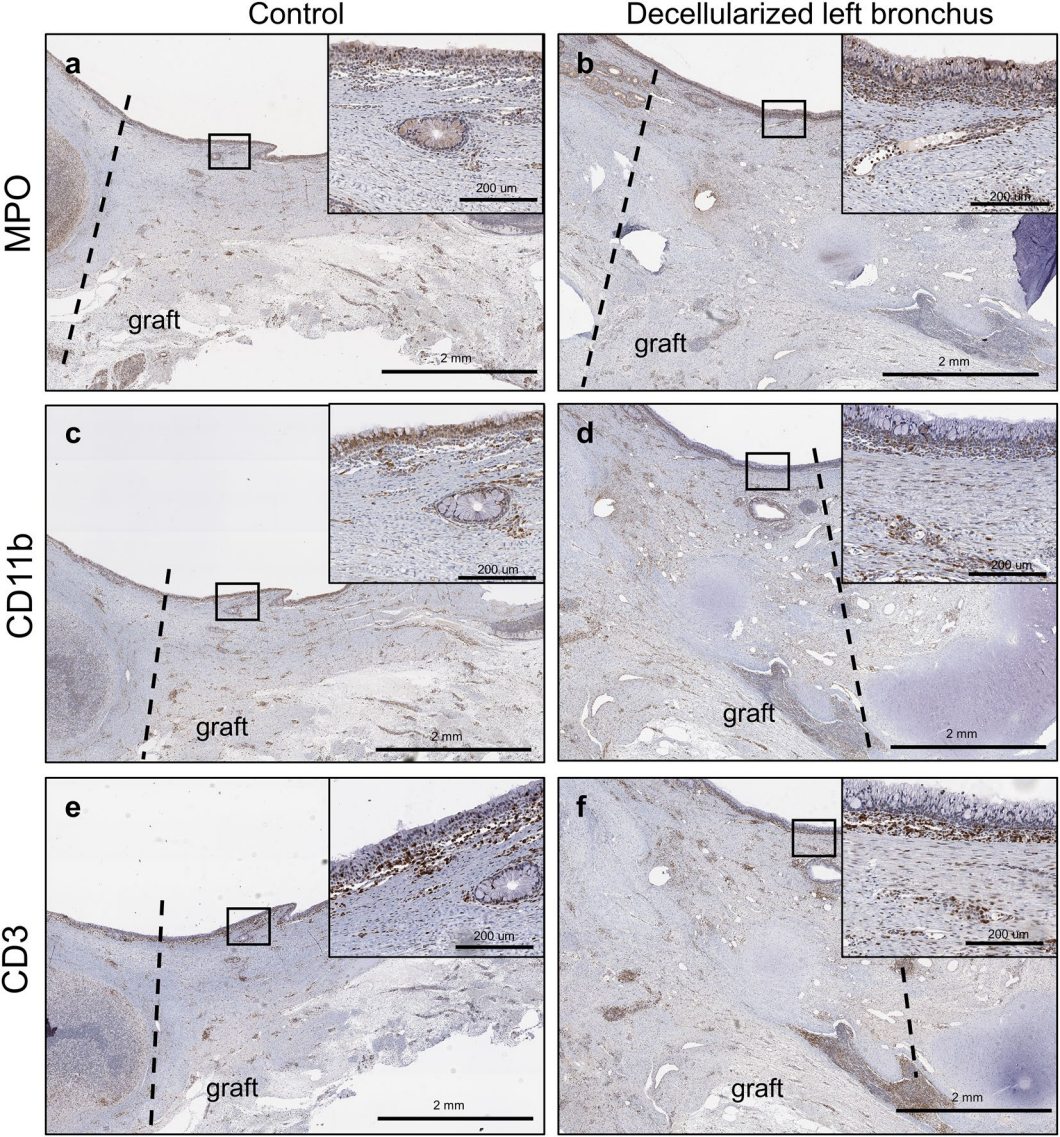
Immunological profile of cells infiltrating the decellularized bronchus. With regards to myeloperoxidase (MPO) positive staining, no difference was observed between the control group and the decellularized bronchus at two months post-operatively (**a**,**b**). Similar findings were observed for CD11b + (**c**,**d**) and CD3 + (**e**,**f**) cells. The infiltrative cells are seen in the submucosal layer of the graft in the decellularized bronchus group (**b**,**d**,**f**), which is similar to the control group (**a**,**c**,**e**) after 2 months. Dotted line represents the junction between the native tissue and the graft.

MPO + (myeloperoxidase) staining showed no difference between the control patch and decellularized bronchus patch at 2 months post-transplant (Fig. 5a,b). Similar findings were observed for CD11b + and CD3 + infiltrating cells in both the control group and the decellularized transplanted tracheal patches (Figs. 5c–f).

### SEM imaging demonstration of re-epithelialization of lumen on both patches

**Figure 6:**
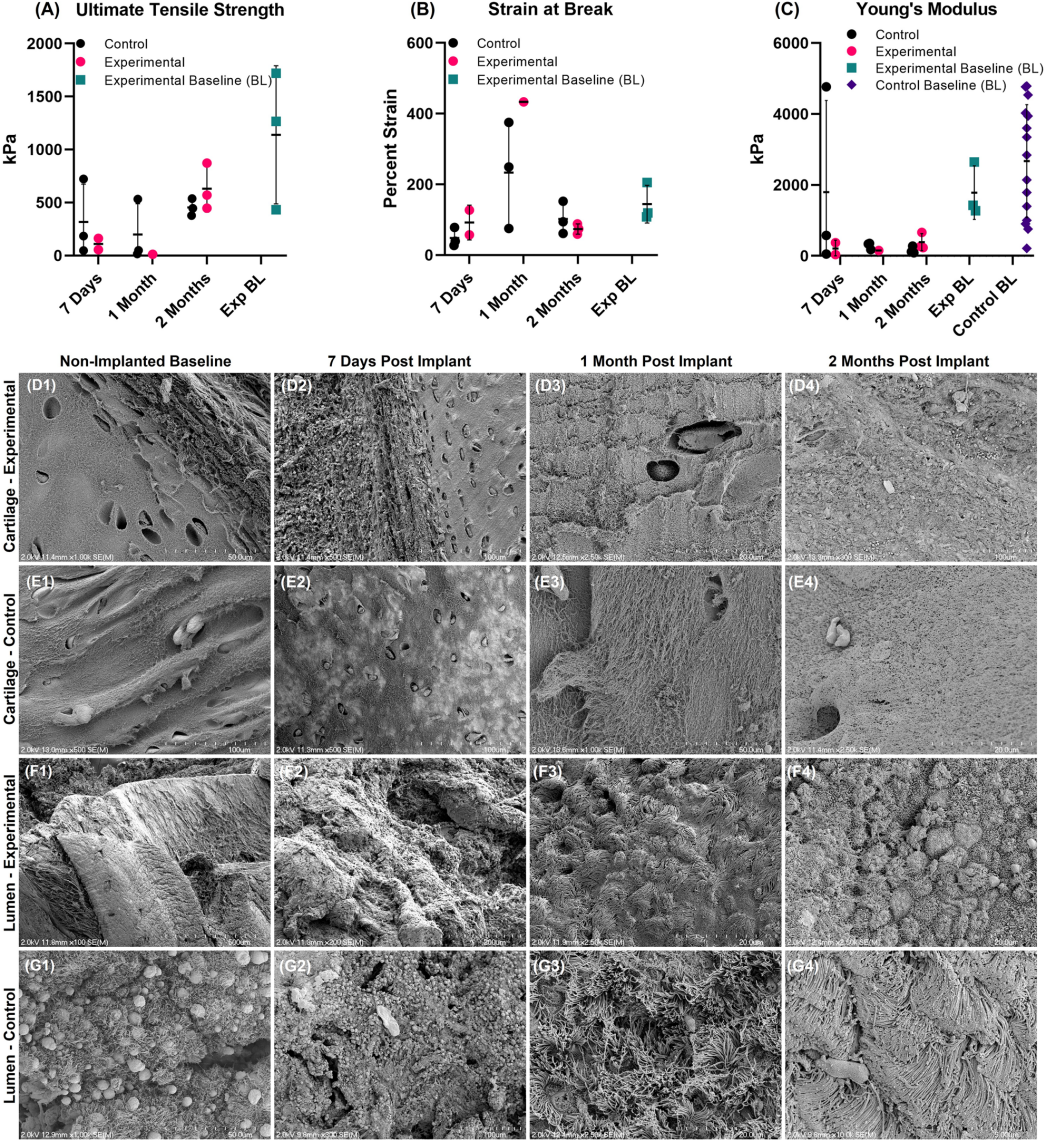
Ultimate tensile strength of both decellularized bronchus and control patches decreased with increasing implantation time through one month, followed by a recovery to near baseline levels after 2 months (**A**). The Young’s modulus decreased for both patch types and did not recover to baseline levels with additional transplantation time (**C**). The strain at break for both patches was similar to their respective controls after 7 days, and increased about four-fold after 1 month, indicating some degree of both control and decellularized bronchus patch degradation (**B**). Strain at break recovered to near baseline levels for both patch types after 2 months (**B**). Observed trends in mechanical data were not significant. Additional replicates are required to corroborate observed trends in mechanical measures. SEM imaging revealed similar structural integrity of cartilaginous tissue in both the control patch (**E2**,**E3**) and the decellularized bronchus patch (**D2**,**D3**) after 7 days and 1 month post transplantation compared to control (**D1**,**E1**). After 2 months, the fiber network in the cartilage of both patches appeared frayed with increased inter-fiber spacing after 2 months (**D4** and **E4**). The lumen of both patches was fully re-epithelialized with ciliated cells after 1 month (**F3** and **G3**) and remained intact after 2 months (**F4** and **G4**).

SEM imaging revealed the presence of ciliated cells on the luminal surface of both the control patches and decellularized bronchus patches after 1 and 2 months (Fig. 6F3,F4,G3 and G4). Noted areas of matted cilia were present on control patch samples after two months (Fig. 6G4). Cross sectional images of patch cartilage did not indicate any evidence of gross degradation after 1 month (Fig. 6D3 and E3 compared to D1 and E1). However, after 2 months, cartilage fibers of both patches appeared frayed with increased inter-fiber spacing, indicating degradation of cartilage in both patches (Fig. 6D4 and E4).

### Strength mechanics decrease through 1 month followed by recovery after 2 months

Although not statistically significant, uniaxial tensile tests demonstrated time dependent changes in ultimate tensile strength (UTS) over the first month for both decellularized bronchus patches and control patches (Fig. 6A), with 1-month patches demonstrating decreased strength relative to 7 day implants. After 2 months, there was a recovery of UTS to near non-transplanted baseline levels. Strain at break (SAB) data corroborates UTS data, revealing largest SAB after 1 month, with 2 months patches approaching non-implanted baseline levels (Fig. 6B). The Young’s modulus also decreased for both patches at all time points, with no recovery to baseline levels (Fig. 6C). Variation of native baseline moduli is attributed to sampling and donor variability. Comparisons among the groups were performed using the Kruskal–Wallis test with Dunn’s post hoc test, which showed no statistically significant difference. Additional replicates are required to statistically corroborate these observed trends in mechanical measures.

## Discussion

Tracheal reconstruction is required in cases of malignancy, trauma and stenosis. Attempts at replacements with artificial materials^8–10,13,16,21^ have led to limited degrees of success^4,11^. Autologous tissues also have limitations such as vascular erosion, risk of infection, limited availability and development of tracheomalacia^27,28^.

Tracheal transplantation represents a possible solution to circumvent these challenges, however the limitations include immune rejection^18,29^ and the use immunosuppressive agents, which can lead to many complications^27,28^. Tissue-engineered approaches yielding a low-immunogenic, biocompatible, and durable construct may serve as an alternative option^1,28,30–32^. Specifically, the use of decellularized tracheal grafts can be useful and possibly negate the need for immunosuppression. These materials are generated using techniques that allow for removal of donor cells and maintenance of the extracellular matrix, therefore reducing and possibly eliminating the need for immunosuppression. Additionally, the use of a bronchus obtained from decellularized lung products has potential application in cases where either airway or lung regeneration is required^33–35^. We investigated the use of a decellularized bronchus obtained from a decellularized lung and evaluated this product in a large animal patch transplantation model. This represents a novel approach using a tissue-engineered product which could have applications in both airway and lung regeneration.

The regeneration of the autologous respiratory epithelium is critical for airway repair in order to prevent exposure of the graft’s luminal surface to external conditions^11,36^. The loss of an epithelial layer leads to granulation hyperplasia which leads to stenosis^37,38^. In this study, the left bronchus from a decellularized porcine lung product served as a tracheal patch for airway transplantation. Our results showed that the decellularized bronchus was well integrated within the native tissue and covered by an epithelial layer as early as 1 month post transplantation which was confirmed by not only immunostaining but also scanning electron microscopy.

Several methods for decellularization have been reported, including chemical agents, biological agents, and physical methods^1,39^. In clinical application, decellularization would remove the allogeneic antigenicity of tracheal tissue from deceased donors, allowing stable engraftment without immunosuppression^1,18,26^. Many studies have shown that incomplete decellularization leads to scaffolds that are eventually degraded and destroyed^3,4,16^. Decellularized patches derived from small intestine submucosa and/or acellular artificial grafts have been also reported. This product can have the native binding sites intact and available for interaction with the recipient’s cells, theoretically increasing the regenerative capacity of these ECMs and minimizing possible inflammatory reactions. However, reinforcement by the artificial material or stenting was required^4,40,41^. We believe that our product could be transplanted without the need for stenting. Particularly interesting in this study, we have shown that this decellularization process leads to maintenance of these grafts used as patches with no differences observed in infiltrating immune cells within the decellularized tracheal patch at 2 months post transplantation with no administration of immunosuppression. Although decellularization has been hypothesized to remove immune cells and antigen presenting cells, there is a lot of literature that shows that there continues to be an active immune response^16^. There are two theoretical possibilities for using this material. One would be in an allogeneic model and the other possibly xenogeneic. Both would require further immunological assessment.

Although our model in this study is not circumferential and does not include long segment transplantation, it represented a proof of concept to test this particular product. Importantly, there have been many complications with other decellularized protocols such as insufficient structural integrity and airway stenosis caused by foreign body inflammatory response due to degradation of the scaffold^15,17,21,28^. We believe that this model can possibly circumvent the challenges previously encountered. We believe further investigation using orthotopic circumferential transplantation will be required to accomplish the clinical needs directly, considering the future of the airway regeneration.

Evaluation of tracheal biomechanics is important in the analysis of decellularized tracheal grafts^15^ and is often used as a measure to predict tracheal collapse. In our study, a decline of the Young’s modulus in native control tissue, as well as the decellularized bronchus grafts, was observed. This is a meaningful observation since even autografts degraded after the temporal resection which shows the inherent difficulty of airway transplantation. However, neither decellularized bronchus grafts nor autografts failed as a part of the tracheal wall in this model. In fact, at 2 months, the tracheal allografts recovered. This fact leads us to consider that partial replacement of airway is possible using this product.

Further long-term studies will be needed for the assessment of decellularized bronchus graft rejection, stenosis, and function for clinical applications. Animals in this model, which is an allogeneic (not xenogeneic) transplant model, survived without symptoms or rejection for two months, which demonstrates that acute rejection is not clinically present despite lack of immunosuppression. Re-epithelialization of this product and long-term outcomes of circumferential transplantation will be required to further study the use of the decellularized bronchus and its clinical applications.

## Conclusion

This study evaluated a decellularized bronchus graft for airway regeneration. The bronchus from a decellularized porcine lung product functioned as an airway patch in a porcine model. Revascularization and re-epithelialization were observed and immunological reactions were comparable to the autograft. This approach is clinically relevant and could potentially be utilized for future applications for lung and tracheal replacements.

### Supplementary Information

Supplementary Figure 1.

## Data Availability

The datasets generated and/or analyzed during the current study are available in the following repository https://figshare.com/s/e45d950170302a1524b1.
