# Supplementary material for: Evaluation of a decellularized bronchial patch transplant in a porcine model
Source: Sci Rep. 2023 Dec 8;13:21773. doi: 10.1038/s41598-023-48643-y (PMC10709302; doi:10.1038/s41598-023-48643-y)
Supplement: Supplementary file 1 — Supplementary Figure 1. [file 41598_2023_48643_MOESM1_ESM.docx]

**
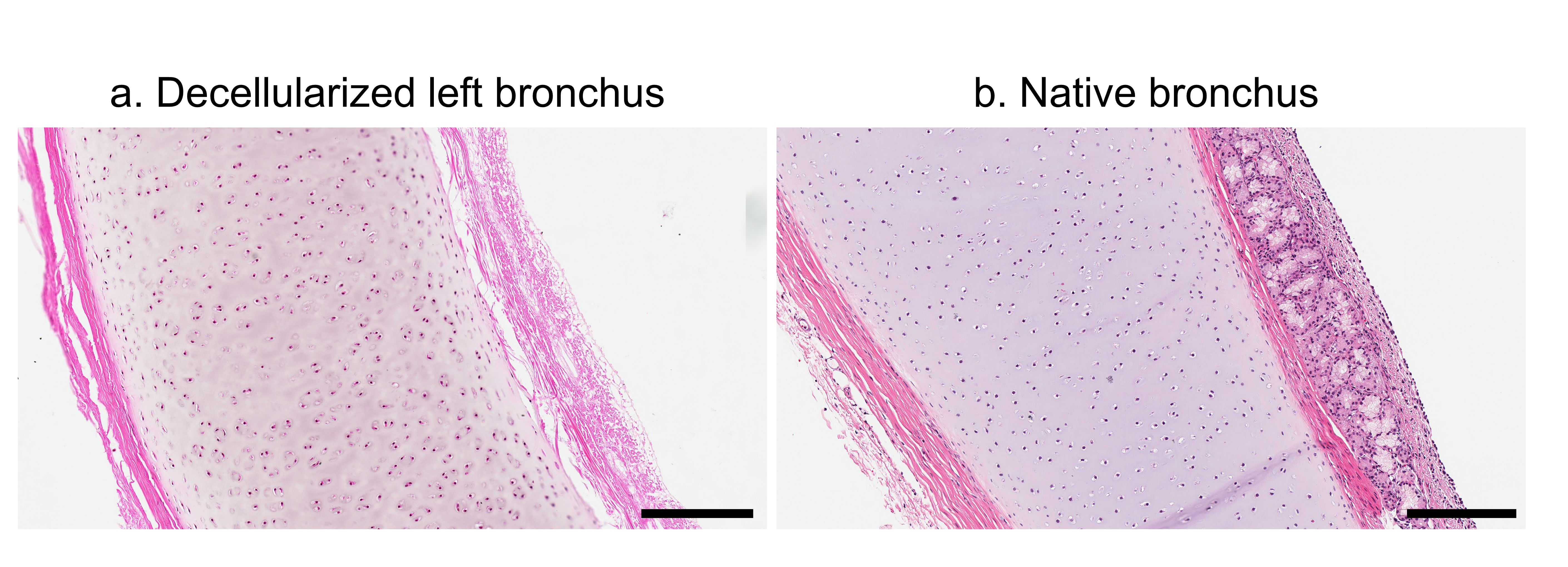
**

**Supplemental Figure 1**. The decellularized bronchus was evaluated histologically by H&E staining, and showed removal of nuclei and cells from the epithelial layer in comparison to the native bronchus (b). (Scale bar = 200um)
